# Rapid Generation of Murine Bispecific Antibodies Using FAST-Ig^TM^ for Preclinical Screening of HER2/CD3 T-Cell Engagers

**DOI:** 10.3390/antib13010003

**Published:** 2024-01-02

**Authors:** Hikaru Koga, Haruka Kuroi, Rena Hirano, Hiroyuki Hirayama, Yoshiaki Nabuchi, Taichi Kuramochi

**Affiliations:** Chugai Pharmaceutical Co., Ltd., Yokohama 244-8602, Japan

**Keywords:** bispecific antibody, FAST-Ig, surrogate mouse bispecific antibody, orthogonal Fab, single-cell production

## Abstract

Bispecific antibodies (BsAbs) can bind to two different antigens, enabling therapeutic concepts that cannot be achieved with monoclonal antibodies. Immuno-competent mice are essential for validating drug discovery concepts, necessitating the development of surrogate mouse BsAbs. In this study, we explored the potential of FAST-Ig^TM^, a previously reported BsAb technology, for mouse BsAb production. We investigated charge-based orthogonal Fab mutations to facilitate the correct assembly of heavy and light chains of mouse antibodies and employed knobs-into-holes mutations to facilitate the heterodimerization of heavy chains. We combined five anti-CD3 and two anti-HER2 antibodies in mouse IgG1 and IgG2a subclasses. These 20 BsAbs were analyzed using mass spectrometry or ion exchange chromatography to calculate the percentages of BsAbs with correct chain pairing (BsAb yields). Using FAST-Ig, 19 out of the 20 BsAbs demonstrated BsAb yields of 90% or higher after simple protein A purification from transiently expressed antibodies in Expi293F cells. Importantly, the mouse BsAbs maintained their fundamental physicochemical properties and affinity against each antigen. A Jurkat NFAT-luciferase reporter cell assay demonstrated the combined effects of epitope, affinity, and subclasses. Our findings highlight the potential of FAST-Ig technology for efficiently generating mouse BsAbs for preclinical studies.

## 1. Introduction

Bispecific antibodies (BsAbs) are capable of binding to two distinct antigens or epitopes, enabling them to exhibit mechanisms of action that cannot be achieved by monospecific antibodies. This unique property has shown promise in treating various diseases, including cancer, hemophilia, and eye diseases [1]. Although several molecular formats for BsAbs have been reported [2], IgG-type BsAbs are preferred formats for therapeutic antibodies due to their stability and lower immunogenicity. However, when IgG-type BsAbs are expressed in a single cell, the random assembly of two heavy chains (HCs) and two light chains (LCs) can result in nine mispairs, except for the correctly assembled BsAb. To promote the correct assembly of human BsAbs, strategies have been developed separately for the HC heterodimerization and for HC/LC pairings. For the interface of HCs, knobs-into-holes (KiH) [3,4] and charge pairs designs [5,6] have been described, while for the HC/LC interfaces, CrossMab [7], Orthogonal Fab (fragment antigen-binding) [8], and DuetMab [9] have been described in several reviews [1,2].

In antibody drug discovery, animal immunization, particularly immunization of mice, is still the primary method for generating new paratopes [10]. Additionally, surrogate mouse antibodies are often required to demonstrate a drug discovery concept for animal studies using mice [11]. Therefore, efficient methods for generating mouse BsAbs are crucial for preclinical studies. However, as mentioned in the following paragraph, there have been a limited number of published reports on the creation of mouse BsAbs.

There are two primary methods reported for generating mouse BsAbs. One example is the controlled Fab arm exchange (cFAE), which efficiently generates mouse BsAbs in mouse IgG1 (mIgG1), mIgG2a, and mIgG2b [12]. However, cFAE requires additional steps beyond affinity purification to obtain BsAbs. These steps involve separately preparing the two parental antibodies, mixing them together, and then placing them under reducing conditions. Following this, the reducing agent is removed, allowing for the formation of BsAbs. The other example is a single-cell expression system that necessitates technologies to facilitate not only the correct assembly of HC/HC but also HC/LC. Strategies such as KiH and charge modification have been reported to promote correct HC heterodimerization in mouse BsAbs [13,14,15]. However, there are few reports on the orthogonal Fab design between HC/LC for mouse antibodies. One example involves applying a charge modification to mIgG2a, which is located at the core of the interface between the constant HC domain 1 (CH1) and the light chain constant region (CL) [16]. It should be noted that this report has limited applications, and that the variable region is derived from a human antibody. Additionally, the application of the DuetMab to mouse Cκ and CrossMab has been reported [13,15]. However, these reports have not compared the correct HC/LC pairing efficiency with and without the introduction of modifications, leaving the extent of the effects of these modifications unclear.

Recently, a novel technology called FAST-Ig (Four-chain Assembly by electrostatic Steering Technology—Immunoglobulin) was developed to promote the correct assembly of HC/HC and HC/LC within a single cell [17]. To promote correct HC and LC assembly, FAST-Ig uses charge-based orthogonal Fab design for human IgGs. However, there are no reports on the application of FAST-Ig for mouse IgGs. This study aimed to investigate whether the orthogonal Fab design of FAST-Ig can be applied to mIgG1 and mIgG2a, the two major types of antibodies often used in mouse studies. To promote HC heterodimerization, KiH mutations, which have already been reported [14,15], were utilized. To confirm the applicability for preclinical screening, new mouse BsAbs comprising known anti-cluster of differentiation 3 (CD3) and anti-human epidermal growth factor receptor 2 (HER2) antibodies were generated using FAST-Ig mutations. The activities of these BsAbs were then compared using the Jurkat NFAT luciferase reporter cell line.

FAST-Ig demonstrated high BsAb production efficiency after simple affinity purification, even for mouse antibodies, without the need for downstream processing. Therefore, FAST-Ig is a useful technology for preclinical BsAb screening and preparing mouse surrogate BsAbs.

## 2. Materials and Methods

### 2.1. Preparation of FAST-Ig Antibodies

Site-directed mutagenesis for FAST-Ig mutations and sub-cloning into mammalian expression vectors was performed using an In-Fusion HD Cloning Kit (Clontech, Takara Bio USA, Inc., San Jose, CA, USA, Z9650N) or NEBuilder HiFi DNA Assembly Master Mix (New England BioLabs, Ipswich, MA, USA, E2621X). Antibodies were transiently expressed in 30 mL of Expi293F (Thermo Fisher Scientific, Waltham, MA, USA, A14527) cells transfected with plasmids encoding HCs and LCs, according to the manufacturer’s instructions (Thermo Fisher Scientific). After 5 days of cultivation, cell culture supernatants containing antibodies were harvested and filtered through a Steriflip-GP 50 mL Express Plus PES 0.22 μm (Merck Millipore, Burlington, MA, USA, SCGP00525). Antibodies were purified by protein A affinity chromatography using an Ab-Capcher MAG2™ (ProteNova, Higashi Kagawa, Japan). In this study, the HC and LC of the anti-HER2 antibody were represented as H1 and L1, respectively, while the HC and LC of the anti-CD3 antibody were represented as H2 and L2, respectively. Regardless of the Fc sequence or the presence or absence of FAST-Ig mutations, the following BsAbs were transiently produced using optimized plasmid mass ratios: m2C4/mHIT3a, m2C4/m12F6, m2C4/mUCHT1, and m2C4/mOKT3 at H1:L1:H2:L2 = 1:1:1:1; m4D5/mHIT3a, m4D5/m12F6, m4D5/mUCHT1, and m4D5/mOKT3 at H1:L1:H2:L2 = 2:2:1:1; m2C4/mSP34 at H1:L1:H2:L2 = 1:1:1:8; and m4D5/mSP34 at H1:L1:H2:L2 = 2:2:1:8 (See Table 1 for the names of the components). The antibodies with low expression levels were combined from multiple batches and concentrated using Amicon Ultra 0.5 mL (Merck Millipore, UFC501024) if necessary. To promote HC heterodimerization, the disulfide-linked knobs-into-holes mutations (dKiHv14), knobs (Y349C, T366W) in the H1, and holes (E356C, T366S, M368A, Y407V) in the H2, were introduced [4]. Also, to abrogate binding to mouse Fc gamma receptors (mFcγRs), the mutations P235K and S239K for mIgG1 [18] and L235R, G236R, and S239K for mIgG2a [19] were introduced.

Homodimeric antibodies used for affinity evaluation and thermal stability were transiently expressed in Expi293F cells and harvested after 4–5 days of cultivation. mIgG1 antibodies were purified by Ab-Capcher MAG2™ (ProteNova) or MonoSpin ProG (GL science, Tokyo, Japan), and mIgG2a were purified by Ab-Capcher MAG2™ (ProteNova) or MonoSpin ProA (GL science) or MonoSpin ProG (GL science).

### 2.2. Antibody Homology Model Generation

The antibody modeling of Fab structures with and without FAST-Ig mutations was performed by using the “Antibody Modeler” implemented in Molecular Operating Environment (MOE) 2022.09. The modeling workflow and methods are described by Maier and Labute [27]. In the case of the modeling of an antibody to which mutations are introduced in the constant region, the sequence alignment with the template might be misaligned by MOE 2022.09. In these cases, alignments were manually adjusted so that mutated positions were correctly aligned before building the structural model.

To create HER2-Fab complex structure models, each modeled antibody structure of m4D5 and m2C4 was superimposed on the extracellular domain of human HER2, using the relative positions of trastuzumab in 1N8Z (PDB: Protein Data Bank) and pertuzumab in 1S78 (PDB), respectively. 

To create CD3-Fab complex structure models, the N-terminal disordered region of the CD3ε structure in 1XIW (PDB) was modeled by “Loop Modeler” (MOE), and the CD3ε N-terminal structure solved in 8F0L (PDB) was superposed on it. Using this structure as a template, the entire structure of CD3ε was modeled by the “Homology Model” (MOE), and then the modeled structure of mSP34 was superimposed on the ADI-26906 antibody in 8F0L (PDB). Modeled antibody structures of mUCHT1 and mOKT3 were superposed on this complex using the relative position of the UCHT1 antibody in 1XIW (PDB) and OKT3 antibody in 1SY6 (PDB). Regarding the distances in the figures, we placed a membrane in the presumed region and estimated distances using an approximate perpendicular line from the centroid of each molecule to the membrane. All molecular graphic images were prepared using CueMol (http://www.cuemol.org accessed on 20 August 2023).

### 2.3. Estimation of Percentage of BsAb by Mass Spectrometry

Mass spectra were obtained using a SYNAPT G2-Si HDMS mass spectrometer equipped with an electrospray ion source and MassLynx data processor (Waters Corp., Milford, MA, USA). Samples were injected into the mass spectrometer via an ACQUITY UPLC I-class system (Waters Corp., Milford, MA, USA). Each sample was diluted to 0.2 mg/mL with 100 mmol/L ammonium hydrogen carbonate followed by PNGaseF digestion (Roche; 11365193001 or New England Biolabs; P6044S), and 3.0 µL was injected into the above LC/MS equipped with a MassPREP Micro Desalting Column, 20 µm, 2.1 mm × 5 mm (Waters Corp., Milford, MA, USA). The samples were eluted by a linear gradient of 20–80% *v*/*v* acetonitrile containing 0.1% *v*/*v* formic acid for 10 min at 0.1 mL/min at 70 °C. For the mass spectrometer, an electrocapillary voltage of 3.0 kV and the ions in the *m*/*z* 600–5000 were obtained. The mass spectra were deconvoluted by MaxEnt1. To estimate the BsAb yield (%), a calculation method proposed by Yin et al. [28] was employed, and the peak intensities used were obtained by the deconvolution spectra.

### 2.4. Estimation of Percentage of BsAb by Cation Exchange Chromatography

To estimate the BsAb yields of m2C4/mUCHT1 and m4D5/mUCHT1 pairs, cation exchange chromatography (CIEX) analysis was carried out using a YMC-BioPro IEX SF HPLC Column (YMC; non-porous, 5 µm, 100 × 4.6 mm, PEEK) on an Alliance HPLC System (Waters) at 40 °C. Buffer A (20 mmol/L sodium acetate, pH 5.0) and Buffer B (20 mmol/L sodium acetate, 1 mol/L NaCl, pH 5.0) were used at a flow rate of 0.5 mL/min. A binary pump delivered a linear gradient of 0–50% Buffer B over 100 min. The column was then washed for 3 min at 100% Buffer B and further equilibrated for 25 min at 0% Buffer B. Elution was monitored by UV absorbance at 280 nm. Each sample was diluted to 0.1 mg/mL with Buffer A, and 100 µL was injected for analysis. If the expression levels of reference samples were too low, samples diluted to less than 0.1 mg/mL were injected. For the BsAb without the FAST-Ig mutations, the chromatograms of H1L1H2L1 and H1L2H2L2 mispair reference samples were used to separate the BsAb chromatograms at the overlapping regions. The area ratio between H1L1H2L1, H1L2H2L2, and the remaining peaks were calculated, and the BsAb yield was determined using a calculation method similar to that used in mass spectrometry [28]. For the BsAb containing the FAST-Ig mutations, the expression levels of H1L1H2L1 and H1L2H2L2 after Protein A purification were below the detection limit of the Lunatic instrument (Unchained Labs) used for protein quantification (<0.03 mg/mL). This indicates that they were barely expressed in the BsAb production system. Therefore, it was concluded that the BsAb yield was approximately 100%.

### 2.5. Surface Plasmon Resonance (SPR)

The binding kinetics of the antibodies against human HER2 and human CD3 were assessed at pH 7.4 at 37 °C using the Biacore T200 (Cytiva, Marlborough, MA, USA). Anti-mouse IgG (Fc) antibody (Mouse antibody capture kit) was immobilized onto all flow cells of a CM4 chip using an amine coupling kit (Cytiva). Anti-HER2 and anti-CD3 antibodies and analytes were prepared in PB-P+ (Na-phosphate buffer 0.05 mol/L, NaCl 0.15 mol/L, 0.05 *w*/*v*% P-20) at pH 7.4. Antibodies were adjusted to concentrations corresponding to 200 RU for assessing binding with recombinant human HER2 (Sino Biological, 10004-H08H) and 630 RU for recombinant human CD3ε-CD3δ heterodimeric protein (CD3εδ) (In-house prepared, Table S1). Human HER2 was prepared by two-fold serial dilutions (6.3 nmol/L to 100 nmol/L). Human CD3 was prepared by two-fold serial dilutions (1.2 nmol/L to 4800 nmol/L) for mUCHT1 and mSP34, (18.8 nmol/L to 19,200 nmol/L) for mHIT3a and mOKT3, and (75 nmol/L to 76,800 nmol/L) for m12F6. The sensor surface was regenerated with Glycine 1.5 with 10 mmol/L glycine–HCl, pH 1.5 (Cytiva). Kinetic parameters were determined by processing and fitting the data to the 1:1 binding model for m4D5, m2C4, mUCHT1, and mSP34 and the steady state model for mHIT3a, mOKT3, and m12F6 using Biacore T200 Evaluation software, version 3.0 (Cytiva, Marlborough, MA, USA).

### 2.6. Size Exclusion Chromatography

SEC was performed using an ACQUITY UPLC H-Class (Waters) HPLC system. Ten µL of samples, diluted to 0.1 mg/mL, were injected onto a TSKgel SuperSW3000 Column, 4 µm, 4.6 mm × 150 mm (Tosoh Bioscience, Tokyo, Japan). A linear gradient of mobile phase A (50 mmol/L sodium phosphate, 300 mmol/L NaCl, pH 7.0) was applied over 10 min at a flow rate of 0.35 mL/min, with UV absorbance monitored at 215 nm. The column compartment temperature was set at 30 °C. For samples with expression levels lower than 0.1 mg/mL, the analysis was performed at their respective concentrations.

### 2.7. Thermal Shift Assay (TSA)

The Tm was determined using a CFX384™ Real-Time System C1000 Touch™ Thermal Cycler (Bio-Rad, Hercules, CA, USA). Briefly, 18 μL of 0.1 mg/mL sample was mixed with 2 μL of 100× Sypro orange protein gel stain (Invitrogen). The plate was ramped from 30.0 °C to 99.6 °C with 0.6 °C/step, and the temperature was held for 2 s for the subsequent step. The Tm was assigned using the first derivative of the raw data. To compare the stability of antibodies before and after the introduction of FAST-Ig mutations, the first peak of the melting curve (Tm1) was referenced.

### 2.8. Jurkat NFAT-Luciferase Reporter Cell Assay

For each anti-HER2/CD3 BsAb, eight serial ten-fold dilutions were prepared, ranging from 30 μg/mL to 3 pg/mL. The assay medium consisted of RPMI 1640 medium (Nakalai Tesque, Inc., Kyoto, Japan) supplemented with MEM non-essential amino acids, Sodium pyruvate, and 10% FBS. Ten μL of antibody solution was added to the wells of white 384 well plates. To analyze target-dependent T cell activation, 20 μL of a 1 to 6 mixture of target cells (BxPC-3) and Jurkat NFAT-luc effector cells at a total cell number of 3.5 × 10^4^ in assay medium was added to each well. To analyze target-independent T cell activation, only 20 μL of Jurkat NFAT-luc effector cells in assay medium at a total cell number of 3.0 × 10^4^ were added to each well. The assay plates were incubated overnight in an incubator at 37 °C and 5% CO_2_. Thirty μL of Bio-Glo luciferase Assay Reagent (Promega) was added to each well. Plates were incubated for 10 min at room temperature before bioluminescence readout using a microplate reader (EnVision, PerkinElmer, Waltham, MA, USA). Each concentration was tested in duplicates on plate 1 and plate 2. The bioluminescence readouts between interplates were normalized by blank wells, and the relative luminescence units (RLU) of duplicates were averaged.

### 2.9. SDS-PAGE

BsAb samples were subjected to non-reducing sodium dodecyl sulfate-polyacrylamide gel electrophoresis (SDS-PAGE) to determine whether the disulfide bonds between HC and LC were correctly formed. Each 1 μg sample was mixed with SDS Sample Buffer Solution (2ME-) (× 4) (Wako) and heated at 95 °C for 2 min, and then loaded onto a Mini-PROTEAN TGX gel (4–20%, 15 well) (Bio-Rad) alongside a molecular weight marker (Precision Plus Protein™ All Blue Prestained Protein Standards, Bio-Rad). Electrophoresis was performed using a Power PAC 3000 (Bio-Rad) at a constant voltage of 200 V for 35 min. The gel was then removed from the cassette and stained with Quick-CBB (Wako), followed by destaining in Milli-Q water. The stained gel was imaged using a ChemiDoc Touch MP (BIO-Rad). 

## 3. Results

### 3.1. Comparison of Mouse and Human Antibody Amino Acid Sequences and Structures at the FAST-Ig Mutation Positions in the HC/LC Interface

**Figure 1:**
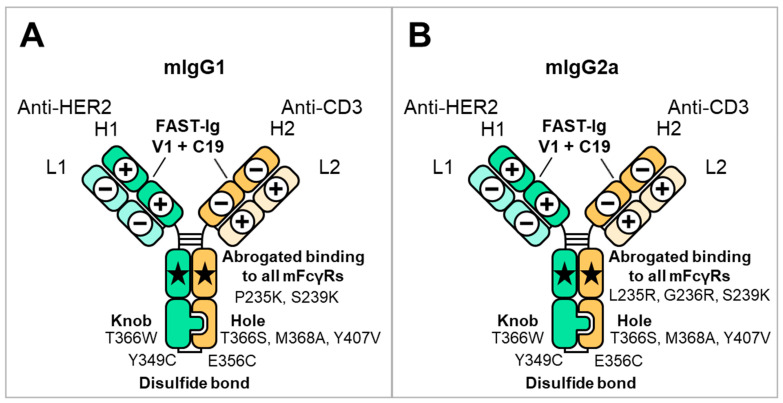
Schematic illustration of mouse BsAbs and the introduced mutations used in this study. The HC and LC of anti-HER2 are represented as H1 and L1, respectively. The HC and LC of anti-CD3 are represented as H2 and L2, respectively. The FAST-Ig V1+C19 mutations are shown in Table 2. To promote HC heterodimerization, the disulfide-linked knobs-into-holes mutations (dKiHv14) were introduced. The numbering of Fc mutations is defined by Eu numbering [29]. (**A**) mIgG1 FAST-Ig format with abrogated binding to all mFcγR. (**B**) mIgG2a FAST-Ig format with abrogated binding to all mFcγR.

In this study, we aimed to examine the applicability of FAST-Ig to mouse antibodies by evaluating mIgG1 and mIgG2a antibodies commonly used in mouse experiments. Although there are multiple versions of FAST-Ig used for human IgG, we focused on the application of V1+C19 amino acid mutations (hereafter shown as V1+C19) [17], which includes mutations in the variable region, to ensure correct HC/LC pairing in mouse antibodies with minimal preliminary examination (Figure 1, Table 2).

**Figure 2:**
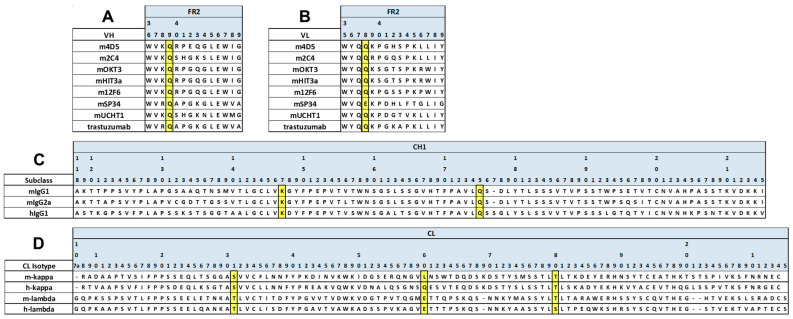
Positions of FAST-Ig mutations in the alignment of representative mouse and human antibody sequences. The sequences of anti-HER2 and anti-CD3 antibodies used in this study are displayed. FAST-Ig mutation positions in framework region 2 (FR2) sequences of VH (**A**) and VL (**B**), as well as CH1 (**C**) and CL (**D**) sequences, are highlighted in yellow. Numbering is defined as shown in the legend of Table 1. For mouse antibodies, gaps were included to conform as closely as possible to Kabat or Eu numbering. human IgG1: hIgG1, mouse kappa: m-kappa, mouse lambda: m-lambda.

First, to infer whether the V1+C19 could be used for mouse antibodies, we aligned and compared representative amino acid sequences of mouse and human antibody variable regions. As a result, we found that the FAST-Ig mutation sites in the variable regions were conserved in both species, except for VL_Glu38 in mSP34 (Figure 2A,B). Similarly, by comparing the CH1 and CL regions of mIgG1 and mIgG2a, we found that the original FAST-Ig residues in the CH1 region were identical to the human sequence (Figure 2C). Additionally, the CL region, except for Leu160 in mouse kappa, was composed of identical or similar amino acid sequences in humans and mice (Figure 2D).

**Figure 3:**
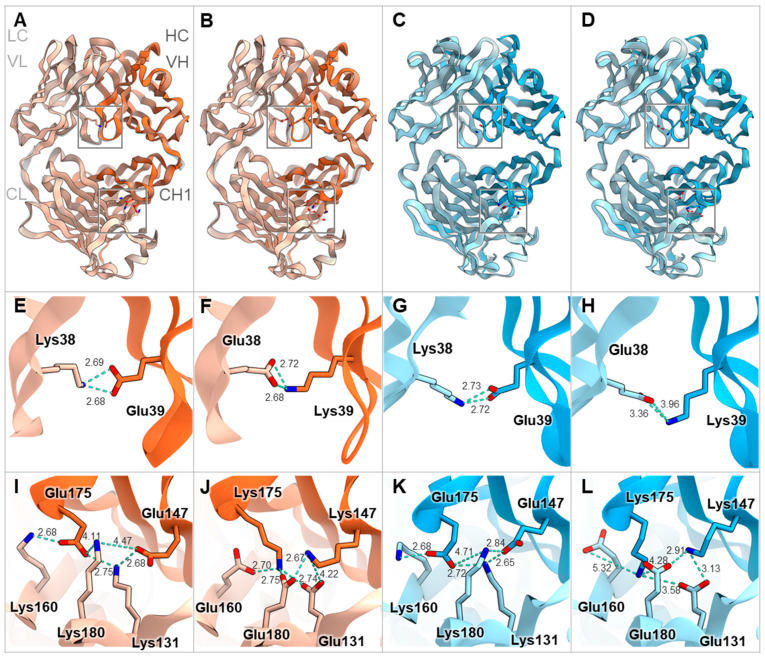
Fab structure models of mIgG1-kappa and mIgG2a-lambda with and without FAST-Ig V1+C19 mutations. Structural superpositions of the Fab of mIgG1-kappa (HC and LC are orange and light orange, respectively) (**A**,**B**) and mIgG2a-lambda (HC and LC are blue and light blue, respectively) (**C**,**D**) containing FAST-Ig V1+C19 mutations and the Fab without FAST-Ig mutations (gray). The key residues are shown as stick models. (**E**–**H**) Close-up views of the charge-pairs of FAST-Ig V1 mutations in (**A**–**D**), respectively. (**I**–**L**) Close-up views of the charge-pairs of FAST-Ig C19 mutations in (**A**–**D**), respectively. The key interactions and distances are shown with light blue dashed lines. For mIgG1-kappa and mIgG2a-lambda, mOKT3 and mSP34 sequences are used, respectively. Each Fab structure’s model was generated by MOE software (described in Materials and Methods). Molecular graphic images were prepared using CueMol (http://www.cuemol.org accessed on 20 August 2023).

Furthermore, we modeled the Fab structures of representative mouse antibodies mOKT3 (mIgG1, kappa) and mSP34 (mIgG2a, lambda) with and without V1+C19 using MOE (Molecular Operating Environment) software. Overlaying each Fab before and after the introduction of the FAST-Ig mutations resulted in a high degree of congruence for all cases (Figure 3A–D). Furthermore, examination of the V1 mutations in the variable region and C19 mutations in the CH1/CL region showed that all residue pairs were within 10 Å, enabling electrostatic interactions (Figure 3E–L).

Taken together, these findings suggest that the V1+C19 of FAST-Ig, based on amino acid sequence and structural modeling, may also be applicable for promoting proper HC/LC pairing in mouse antibodies.

### 3.2. Application of V1+C19 to Anti-HER2/CD3 Bispecific Antibodies

The anti-HER2/CD3 BsAb has been reported as a potential attractive T cell bispecific antibody for solid tumors expressing HER2 [31,32]. Therefore, we investigated the applicability of the V1+C19 to mouse BsAb targeting HER2 and CD3. Two types of anti-HER2 antibodies, m4D5 and m2C4, and five types of anti-CD3 antibodies, mOKT3, mHIT3a, m12F6, mSP34, and mUCHT1, were used (Table 1). We applied the ten combinations of these antibodies to the mIgG1 and mIgG2a subclasses. As shown in Figure 1, the HC and LC of anti-HER2 antibodies are designated as H1 and L1, while those of anti-CD3 antibodies are designated as H2 and L2, respectively. The disulfide-linked knobs-into-holes mutations (dKiHv14) (knobs: Y349C, T366W in H1, and holes: E356C, T366S, M368A, Y407V in H2) were incorporated into the constant region of these HCs to promote HC heterodimerization [4]. To inhibit binding to mFcγRs, the amino acid mutations P235K and S239K for mIgG1 [18] and L235R, G236R, and S239K for mIgG2a [19] were introduced.

**Figure 4:**
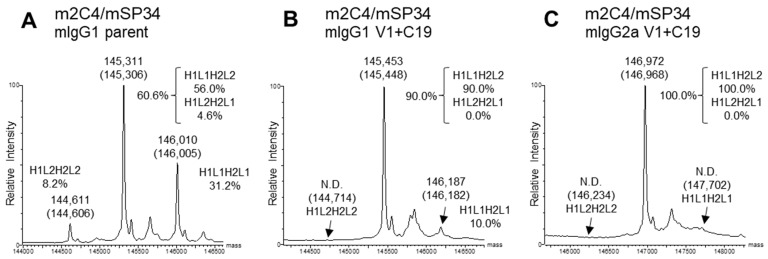
Typical deconvoluted mass spectra and percentages of IgG species. The percentage of H1L1H2L2 was estimated by a method proposed in a previous report [28]. Theoretical masses are denoted in parentheses. Each spectrum shows the results of m2C4/mSP34 (**A**) mIgG1 parent; (**B**) mIgG1 V1+C19; (**C**) mIgG2a V1+C19. All antibodies in the figure include the dKiHv14 mutations shown in Figure 1, as well as mutations that suppress mFcγR binding. Antibodies without the FAST-Ig V1+C19 mutations are denoted as “parent”. N.D.: Not detected.

For the expression of the BsAbs, transfection was performed using optimized plasmid ratios for each set of 4 BsAb genes. The optimized plasmid ratios were set such that the expression levels of each original homodimeric antibody (H1L1H1L1 or H2L2H2L2), which lacks dKiHv14 mutations, were as equal as possible when the HC and LC ratios were varied. The percentage of target BsAb (BsAb yield) of the antibodies purified with Protein A was estimated by previously reported methods, using mass spectra [28]. In this analysis, the antibodies were enzymatically deglycosylated to remove the N-glycan at Asn297 in the Fc region. For mIgG1, most of the glycans were completely removed, so we used the calculated spectrum corresponding to the theoretical mass of the deglycosylated form (Figure 4A,B). However, particularly for mIgG2a, some residual Fc fragments with partially cleaved glycans remained. Therefore, we calculated the BsAb yield using only the spectra of the completely deglycosylated form (Figure 4C). Due to the introduction of the dKiHv14, mispaired antibodies, including HC-homodimerization, were confirmed to be almost negligible. The mass spectrum ratios of H1L1H2L2 (H1L2H2L1), H1L1H2L1, and H1L2H2L2 were calculated, and the percentage of H1L1H2L2 was determined. As a result, all BsAbs with V1+C19 in the mIgG1 subclass demonstrated BsAb yields of 90.0% or higher, achieving comparable or higher BsAb yields than the parent antibodies (In this paper, the term “Parent antibodies” refers to BsAbs that have the dKiHv14 and mFcγR binding-suppressing mutations as described in Figure 1 but do not contain the FAST-Ig V1+C19 mutations). Additionally, in the mIgG2a subclass, the incorporation of FAST-Ig led to BsAb yields of more than 81.0% for all BsAbs (Table 3). For m4D5/mUCHT1 and m2C4/mUCHT1, the theoretical mass of the target BsAb and other mispairings was too close (<30 Da difference) to calculate the BsAb yield using mass spectrometry. Therefore, these antibodies were analyzed by the area ratio of CIEX. Although the BsAb yield of parent antibodies could be calculated, no mispaired standard samples of H1L1H2L1 and H1L2H2L2 were expressed for antibodies with V1+C19, suggesting a virtually 100% BsAb yield (Figure S1).

Thus, the FAST-Ig V1+C19 has been demonstrated to be applicable for promoting the correct pairing of the HC/LC interface in mouse antibodies.

### 3.3. Retention of Parental Physicochemical Properties in Mouse BsAbs with V1+C19

Next, we confirmed that the introduction of V1+C19 in the antibodies did not significantly alter their basic physicochemical properties compared to the parent antibodies. First, non-reducing SDS-PAGE was performed for each prepared BsAb, confirming that the antibodies with V1+C19 also formed disulfide bonds between the HC and LC (Figure S3). Furthermore, all antibodies were over 95% monomeric by SEC analysis (Figure S4). In addition, SPR analysis demonstrated that even with the V1+C19, the antigen-binding affinity was largely retained at the level of the original antibodies (Table 4, Figure S5). The anti-HER2 antibodies, m4D5 and m2C4, both exhibited strong affinities with *K*_D_ values of approximately 0.8–1.4 nmol/L, while the anti-CD3 antibodies, m12F6, mHIT3a, mOKT3, mSP34, and mUCHT1, each showed a wide range of affinities from approximately *K*_D_ = 16 µmol/L to 1 nmol/L. Moreover, the affinities did not significantly differ between mIgG1 and mIgG2a. Subsequently, the antibodies’ thermal stability was evaluated by determining their Tm using a thermal shift assay. By introducing V1+C19, the Tm of all samples were comparable or decreased within 3 °C, except for m4D5 IgG2a, m4D5 IgG1, m2C4 IgG2a, and mSP34 IgG2a, which decreased by 6.0, 3.6, 3.6, and 3.6 °C, respectively. Despite these changes, all samples maintained a high Tm of over 60 °C, indicating sufficient stability for practical use (Table 5).

### 3.4. Comparative Analysis of Target-Dependent T Cell Activation by HER2/CD3 BsAbs

**Figure 5:**
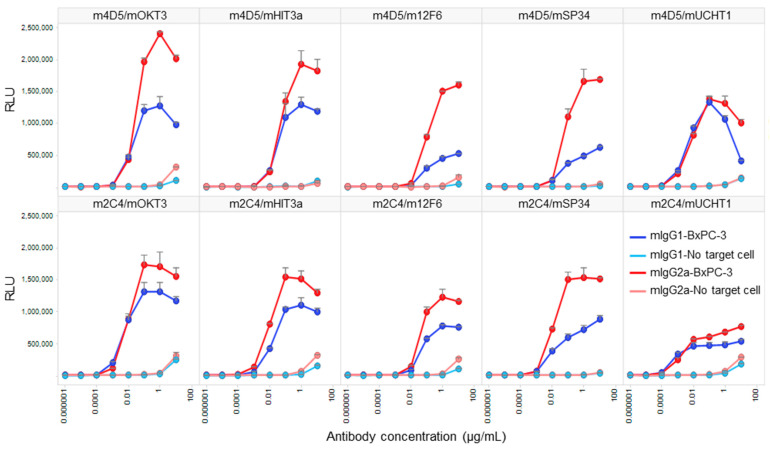
In vitro T cell activation induced by each anti-HER2/CD3 BsAb with FAST-Ig mutations, with or without target cells. Jurkat/NFAT-luc cells were co-incubated with human HER2-expressing BxPC-3 cells and serial dilutions of anti-HER2/CD3 mouse BsAbs with V1+C19 mutations in Table 3. Similar results were obtained in two independent assays. Data shown represent mean + SD for two replicates in a single assay. RLU: relative luciferase unit.

We conducted a Jurkat NFAT-luciferase reporter cell assay to compare the target-dependent T cell activation activity of HER2/CD3 BsAbs generated using V1+C19. To confirm that the activity was target-dependent, we performed the assay under conditions with and without the presence of HER2-expressing cells (BxPC-3). As a result, all BsAbs exhibited strong T cell activation only in the presence of target cells, with minimal activity observed at the highest antibody concentration of 10 µg/mL in the absence of target cells (Figure 5). In all antibody combinations, mIgG2a showed stronger T cell activation than mIgG1, and the anti-HER2 Fab exhibited higher activity for m4D5 than m2C4. The m4D5/mOKT3 IgG2a combination showed the highest maximum T cell activation among all combinations, while the lowest activity was observed for m4D5/m12F6 IgG1 and m2C4/mUCHT1 IgG1. These results demonstrate that by using FAST-Ig and simple affinity purification of mouse BsAbs from transient expression systems, we can effectively compare the potential of various T cell BsAbs combinations in terms of epitopes and subclasses. 

## 4. Discussion

In this study, we successfully applied the FAST-Ig technology, initially developed for human antibodies [17], to efficiently create mouse BsAbs for preclinical research. Two main methods have been reported for generating mouse BsAbs: the cFAE method [12], which reconstitutes two parental antibodies prepared from separate cells, and another method that uses amino acid substitution for correct HC/HC and HC/LC pairing in a single cell [13]. Compared to the cFAE method, FAST-Ig offers advantages as a single-cell expression method, enabling simpler BsAb preparation via affinity purification and removing the need for extra downstream operations.

Among the various FAST-Ig versions, we consistently used the V1+C19, which includes mutations in both the variable and constant regions (Table 2). This version was chosen to effectively achieve high BsAb yields for non-clinical mouse research rather than for clinical development despite the potential reductions in expression levels. As expected, BsAbs with V1+C19 resulted in significantly high BsAb yields for both mIgG1 (≥90.0%) and mIgG2a (≥81.0%), the two main antibody subclasses used in mouse studies (Table 3). Some of the parent BsAbs, such as m4D5/mHIT3a and m2C4/mHIT3a, initially exhibited high BsAb yields exceeding 96%, which could be attributed to the strong preference for cognate HC/LC pairing in the parent antibodies [8,16,17,28,33]. Since the batches of parent antibodies and BsAbs with FAST-Ig differ, it is difficult to strictly compare expression levels in this study. However, for BsAbs, including m4D5 and mSP34 with low parent antibody expression, their expression levels further decreased due to the introduction of FAST-Ig V1+C19, necessitating the concentration of multiple lots for experimental use (data not shown). In the actual antibody screening, maintaining a certain level of parent antibody expression is also essential for effectively completing the process.

To achieve high BsAb yields in a single cell, it is important not only to strongly promote correct inter-chain associations but also to ensure equal expression of both pairs of antibodies per a BsAb [13,16,17,28]. Hence, adjusting the H1:L1:H2:L2 plasmid ratio during transfection is preferable, particularly for antibodies with low expression levels, such as the m4D5 and mSP34. For the m4D5, the amounts of HC-plasmids (H1) used were twice that of all other anti-CD3 antibodies (H2). Notably, due to the extremely low original antibody expression level of mSP34, it was necessary to transfect eight times more LC than HC to yield an appropriate antibody amount. As a result, comparable or higher BsAb yields were obtained for all antibodies compared to parent antibodies (Table 3). However, to increase efficiency, future improvements are needed, such as incorporating information on antibody expression levels during the initial mouse antibody binder screening and adjusting the appropriate plasmid ratio from the outset. 

For the quantification of BsAbs, we used the previously reported mass spectrometry method [28]. However, when dealing with antibodies like m4D5/mUCHT1 and m2C4/mUCHT1, where the molecular weights of the mispaired antibodies are close to those of the correctly paired antibodies, accurate analysis was not possible. Therefore, it was necessary to use other analytical methods, such as CIEX, to verify the BsAb yields (Figure S1). The mass spectrometry-based BsAb yield for m2C4/mOKT3 mIgG2a (81.0%) was higher than that of the parent antibody (51.1%) but lower than other FAST-Ig V1+C19-introduced samples (Table 3). This value may be influenced by mass spectrometry artifacts, such as an elevated baseline, which can overestimate the peak height of a mispaired antibody (Figure S2). Consequently, the true BsAb yield might be higher than these values. Developing a simple and accurate method to calculate BsAb yield for any BsAb pair remains an important challenge in BsAb research.

Our study confirmed that mouse BsAbs generated using FAST-Ig technology are sufficient for screening T cell bispecific engagers using HER2-expressing cells and Jurkat NFAT cells (Figure 5). We used antibodies in both mIgG2a and mIgG1 subclasses that suppress binding to mFcγR to prevent T cell activation independent of target cells. The affinities for HER2 and CD3 were almost equivalent between the two subclasses. The anti-HER2 antibodies, m4D5 and m2C4, showed high affinities for HER2 (*K*_D_ = 0.8–1.4 nmol/L), while the anti-CD3 antibodies had a wide range of affinities (*K*_D_), from 16 µmol/L to 1 nmol/L (Table 4). Therefore, the observed differences in T cell activation between the tested antibodies are likely due to epitope combinations, the distance between the two antigens depending on the subclass, or the affinity for CD3.

**Figure 6:**
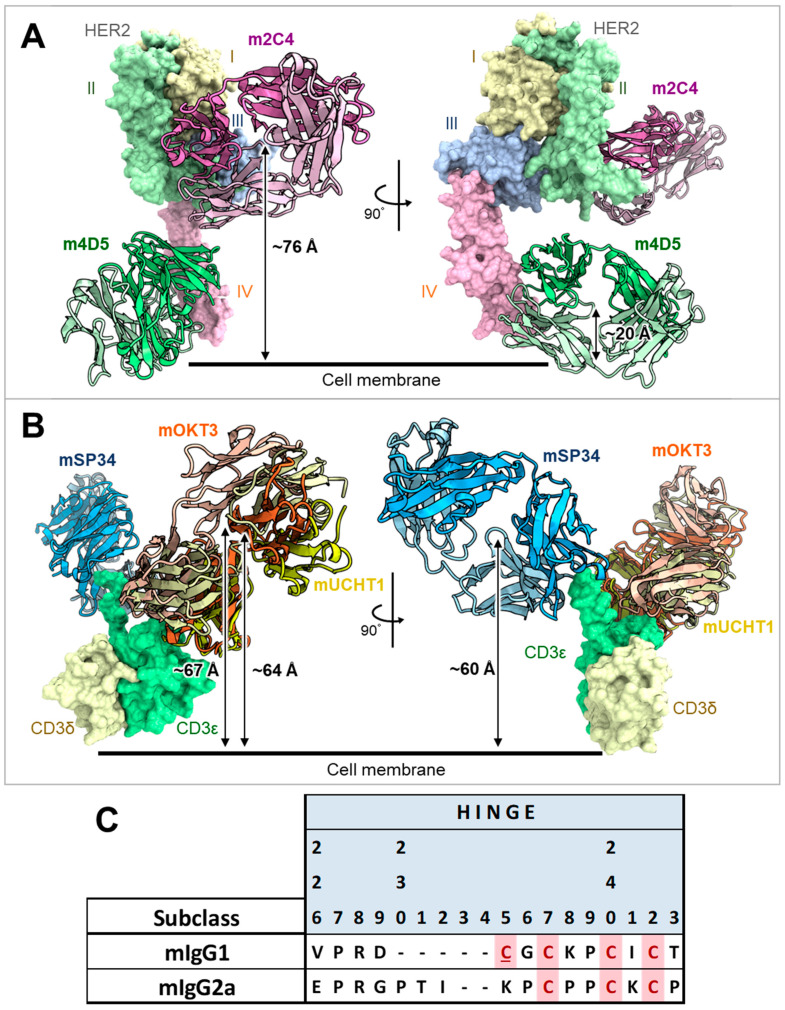
Estimated distances of each anti-HER2 or anti-CD3 Fab from the cell membrane and alignment of hinge sequences of mIgG1 and mIgG2a. (**A**) Complex structure model of m4D5 and m2C4 Fabs with human HER2. The distances between the centroids of m4D5 and m2C4 and the cell membrane were ~20 Å and ~76 Å, respectively. I–IV represent the extracellular domains (ECDs) of human HER2. ECD I: 23–198 (light yellow), ECD II: 199–340 (light green), ECD III: 341–508 (light blue), ECD IV: 509–652 (light pink) in the UniProt database (accession number P04626) [41]. m4D5-Fab (HC: pink, LC: light pink), m2C4-Fab (HC: green, LC: light green). (**B**) Complex structure model of mOKT3, mUCHT1, and mSP34 Fabs with human CD3εδ. The distances between the centroids of mOKT3, mUCHT1, and mSP34 and the cell membrane were ~67 Å, ~64 Å, and ~60 Å, respectively. human CD3ε: light green, human CD3δ: light yellow, mOKT3-Fab (HC: orange, LC: light orange), mUCHT1-Fab (HC: yellow, LC: light yellow), mSP34-Fab (HC: blue, LC: light blue). Refer to materials and methods for model construction and distance calculation in A and B. (**C**) Alignment of amino acid sequences in the hinge region of mIgG1 and mIgG2a. Cys residues involved in disulfide bond formation between HCs are highlighted in pink. Cys residues involved in disulfide bond formation between HC and LC are highlighted in pink and underscored. Residue numbers are defined according to Kabat numbering [30].

The humanized forms of m4D5 and m2C4 antibodies are known as trastuzumab and pertuzumab, respectively [20,21]. Therefore, the epitopes for each are thought to be identical to those of trastuzumab and pertuzumab [34]. This means that m4D5 binds to the membrane-proximal domain IV of HER2 (~20 Å), while m2C4 binds to the membrane-distal domain II (~76 Å) (Figure 6A). On the other hand, the epitopes on CD3εδ for mOKT3 and mUCHT1 have been reported in complex structures and are known to be nearly identical [35]. Furthermore, m12F6 has been reported to have a competing epitope with mOKT3 [24], and their CDR sequences are very similar (Table 1). Moreover, the CDR sequences of mHIT3a are identical to those of mOKT3 (Table 1), suggesting that mOKT3, m12F6, and mHIT3a share the same epitope. mSP34 has been reported to recognize the N-terminal peptide of CD3ε [36], and a binding model of mSP34 and CD3ε was created by referring to the complex structure of ADI-26906 and the N-terminal CD3ε peptide, which exhibits a similar recognition mechanism [37]. As a result, it was inferred that all five anti-CD3 Fabs are located at nearly the same distance of 60–67 Å from the membrane (Figure 6B). In other words, the distance of the immune synapse formed by the combination of HER2 and CD3 epitopes is likely dependent on the HER2 epitopes. In this study, all combinations of antibodies (mIgG2a) showed higher maximum activity with m4D5 than with m2C4 (Figure 5). This is thought to be due to the use of the IgG format, which allows for a relatively larger distance between the two Fabs compared to fragment-type antibodies. This enables m4D5, which binds to the membrane-proximal region, to form an optimal distance for T cell activation. This result is consistent with previous reports that have investigated the distance between various antigen/CD3 pairs using the IgG format [38,39]. 

On the other hand, mIgG2a demonstrated higher activity than mIgG1 despite its hinge being three amino acids longer than that of mIgG1 (Figure 6C). In a previous report comparing the influence of antibody subclasses on the cytotoxic activity of anti-CD19/CD3 BsAbs, it was found that selecting the more flexible hIgG1 subclass, as opposed to the rigid hIgG2 subclass, led to higher activity [40]. Therefore, it is possible that in the case of the anti-HER2/CD3 BsAbs, the more flexible subclasses may have contributed to a more efficient T cell activation.

Regarding CD3 affinity, higher maximum activity was observed with the moderate-affinity antibody mOKT3 (*K*_D_ = 4.5–7.7 µmol/L) than with the higher-affinity antibodies (mSP34: *K*_D_ = 26–57 nmol/L, mUCHT: *K*_D_ = 1.3 nmol/L) (Table 4). Furthermore, among the antibodies targeting the same CD3 epitope (mOKT3, mHIT3a, m12F6), slightly reduced activity was observed, corresponding to the decrease in affinity (*K*_D_) from 4.5–7.7 µmol/L to 16 µmol/L. These findings suggest that a combination with a high-affinity anti-CD3 Fab does not necessarily result in higher T cell activation. Based on previous reports, reduced CD3 affinity could also be effective for better pharmacokinetics and reduced cytokine release [39,42]. In the screening of T cell-engaging BsAbs, it is important to consider the selection of epitope combinations, antibody subclass, and exploration of the optimal affinity range.

In summary, we have shown that FAST-Ig technology can also be used to efficiently express mouse BsAbs. As one application, we have compared the potential of various T cell-engaging BsAbs. This successful application has significance for antibody screening in mouse studies and potential in vivo applications, which can contribute to the preclinical drug discovery process in the BsAb field.

## Figures and Tables

**Table 1:** VH and VL of the original mouse antibody sequences ^1^.

Name	Antigen	CL	VH	VL
m4D5 [20]	HER2	kappa	QVQLQQSGPELVKPGASLKLSCTASGFNIK**DTYIH**WVKQRPEQGLEWIG**RIYPTNGYTRYDPKFQD**KATITADTSSNTAYLQVSRLTSEDTAVYYCSR**WGGDGFYAMDY**WGQGASVTVSS	DIVMTQSHKFMSTSVGDRVSITC**KASQDVNTAVA**WYQQKPGHSPKLLIY**SASFRYT**GVPDRFTGSRSGTDFTFTISSVQAEDLAVYYC**QQHYTTPPT**FGGGTKVEIK
m2C4 [21]	HER2	kappa	EVQLQQSGPELVKPGTSVKISCKASGFTFT**DYTMD**WVKQSHGKSLEWIG**DVNPNSGGSIYNQRFKG**KASLTVDRSSRIVYMELRSLTFEDTAVYYCAR**NLGPSFYFDY**WGQGTTLTVSS	DTVMTQSHKIMSTSVGDRVSITC**KASQDVSIGVA**WYQQRPGQSPKLLIY**SASYRYT**GVPDRFTGSGSGTDFTFTISSVQAEDLAVYYC**QQYYIYPYT**FGGGTKLEIK
mOKT3 [22]	CD3	kappa	QVQLQQSGAELARPGASVKMSCKASGYTFT**RYTMH**WVKQRPGQGLEWIG**YINPSRGYTNYNQKFKD**KATLTTDKSSSTAYMQLSSLTSEDSAVYYCAR**YYDDHYCLDY**WGQGTTLTVSS	QIVLTQSPAIMSASPGEKVTMTC**SASSSVSYMN**WYQQKSGTSPKRWIY**DTSKLAS**GVPAHFRGSGSGTSYSLTISGMEAEDAATYYC**QQWSSNPFT**FGSGTKLEIN
mHIT3a [23]	CD3	kappa	QVQLQESGAELARPGASVKMSCKASGYTFT**RYTMH**WVKQRPGQGLEWIG**YINPSRGYTNYNQKFKD**KATLTTDKSSSTAYMELTRLTSEDSAVYYCAR**YYDDHYCLDY**WGQGTTVTVSS	DIELTQSPAIMSASPGEKVTMTC**SASSSVSYMN**WYQQKSGTSPKRWIY**DTSKLAS**GVPARFSGSGSGTSYSLTISGMEAEDAATYYC**QQWSSNPFT**FGSGTKLELK
m12F6 [24]	CD3	kappa	QVQLQQSGAELARPGASVKMSCKASGYTFT**SYTMH**WVKQRPGQGLEWIG**YINPSSGYTKYNQKFKD**KATLTADKSSSTAYMQLSSLTSEDSAVYYCAR**WQDYDVYFDY**WGQGTTLTVSS	QIVLSQSPAILSASPGEKVTMTC**RASSSVSYMH**WYQQKPGSSPKPWIY**ATSNLAS**GVPARFSGSGSGTSYSLTISRVEAEDAATYYC**QQWSSNPPT**FGGGTKLETK
mSP34 [25]	CD3	lambda	EVQLVESGGGLVQPKGSLKLSCAASGFTFN**TYAMN**WVRQAPGKGLEWVA**RIRSKYNNYATYYADSVKD**RFTISRDDSQSILYLQMNNLKTEDTAMYYCVR**HGNFGNSYVSWFAY**WGQGTLVTVSA	QAVVTQESALTTSPGETVTLTC**RSSTGAVTTSNYAN**WVQEKPDHLFTGLIGGTNKRAPGVPARFSGSLIGDKAALTITGAQTEDEAIYFC**ALWYSNLWV**FGGGTKLTVL
mUCHT1 [26]	CD3	kappa	EVQLQQSGPELVKPGASMKISCKASGYSFT**GYTMN**WVKQSHGKNLEWMG**LINPYKGVSTYNQKFKD**KATLTVDKSSSTAYMELLSLTSEDSAVYYCAR**SGYYGDSDWYFDV**WGQGTTLTVFS	DIQMTQTTSSLSASLGDRVTISC**RASQDIRNYLN**WYQQKPDGTVKLLIY**YTSRLHS**GVPSKFSGSGSGTDYSLTISNLEQEDIATYFC**QQGNTLPWT**FAGGTKLEIK

^1^ VH: heavy chain variable region, VL: light chain variable region. m4D5 and m2C4 are the original mouse antibodies of trastuzumab and pertuzumab, respectively. For the VL of m4D5, an N65S mutation (underlined in the table; Ser65 is the same residue as trastuzumab) is introduced to remove the glycosylation site for easier analysis. The sequences of CDR1-3 within VH and VL are displayed in bold and red.

**Table 2:** FAST-Ig V1+C19 mutations used for mIgG1 and mIgG2a BsAbs ^1^.

Chain Name	V1+C19 Mutations [17]	
Anti-HER2 HC (H1)	VH	CH1
	Q39K	K147/Q175K
Anti-HER2 LC (L1)	VL	CL
	Q38E	S131E/Q160E/T180E
Anti-CD3 HC (H2)	VH	CH1
	Q39E	K147E/Q175E
Anti-CD3 LC (L2)	VL	CL
	Q38K (E38K)	S131K/L160K/T180K (T131K/E160K/T180K)

^1^ Numbering of CH was defined by Eu numbering [29]. VH, VL, and CL were defined by Kabat numbering [30]. For the lambda LC of mSP34, the amino acid mutations listed in parentheses were used.

**Table 3:** BsAb yields for each BsAb pair with different subclasses and presence or absence of FAST-Ig mutations ^1^.

Subclass	FAST-Ig	BsAb Yield (%)									
		m4D5/mOKT3	m4D5/mHIT3a	m4D5/m12F6	m4D5/mSP34	m4D5/mUCHT1	m2C4/mOKT3	m2C4/mHIT3a	m2C4/m12F6	m2C4/mSP34	m2C4/mUCHT1
mIgG1	-	31.5	96.2	72.9	15.1	58.7 *	51.1	98.2	49.0	56.0	21.9 *
mIgG1	V1+C19	100.0	94.5	100.0	100.0	100.0 *	100.0	100.0	98.6	90.0	100.0 *
mIgG2a	V1+C19	100.0	100.0	100.0	100.0	100.0 *	81.0 **	100.0	100.0	100.0	100.0 *

^1^ All BsAbs listed in this table include the dKiHv14 mutations shown in Figure 1 and mutations that suppress mFcγR binding. When the FAST-Ig V1+C19 mutations are not present, it is denoted by “-”. * Due to the close theoretical masses of BsAb and mispairs (H1L1H2L1, H1L2H2L2), the BsAb yields were calculated based on the area ratio in CIEX analysis (Figure S1). BsAbs with FAST-Ig V1+C19 were considered to have a virtually 100% BsAb yield, as no standard samples of H1L1H2L1 and H1L2H2L2 were expressed. ** The calculated BsAb yield was lower than those of other BsAbs with FAST-Ig; this may be due to overestimation of the intensity of H1L1H2L1 (Figure S2).

**Table 4:** Affinities for each anti-HER2 or anti-CD3 Fab with different subclasses and the presence or absence of FAST-Ig mutations ^1^.

Subclass	FAST-Ig	*K*_D_ (mol/L)						
		Anti-HER2		Anti-CD3				
		m4D5	m2C4	mOKT3	mHIT3a	m12F6	mSP34	mUCHT1
mIgG1	-	1.03 × 10^−9^	7.59 × 10^−10^	4.82 × 10^−6^	7.54 × 10^−6^	1.68 × 10^−5^	2.09 × 10^−8^	1.45 × 10^−9^
mIgG1	V1+C19	1.27 × 10^−9^	7.84 × 10^−10^	4.48 × 10^−6^	8.32 × 10^−6^	1.61 × 10^−5^	5.69 × 10^−8^	1.32 × 10^−9^
mIgG2a	-	1.14 × 10^−9^	1.08 × 10^−9^	6.58 × 10^−6^	1.04 × 10^−5^	1.67 × 10^−5^	1.60 × 10^−8^	1.02 × 10^−9^
mIgG2a	V1+C19	9.60 × 10^−10^	1.36 × 10^−9^	7.68 × 10^−6^	1.04 × 10^−5^	1.64 × 10^−5^	2.57 × 10^−8^	1.32 × 10^−9^

^1^ All antibodies listed in this table are homodimeric antibodies composed of a single HC and a single LC. They include the mFcγR binding-suppressing mutations shown in Figure 1 but do not contain the dKiHv14 mutations. When the FAST-Ig V1+C19 mutations are not present, it is denoted by “-”.

**Table 5:** Thermal stabilities (Tm1) determined by TSA for each anti-HER2 or anti-CD3 Fab with different subclasses and the presence or absence of FAST-Ig mutations ^1^.

Subclass	FAST-Ig	Tm1 (°C)						
		Anti-HER2		Anti-CD3				
		m4D5	m2C4	mOKT3	mHIT3a	m12F6	mSP34	mUCHT1
mIgG1	-	72.6	75.6	75.6	73.2	76.2	67.2	75.0
mIgG1	V1+C19	69.0	74.4	75.0	72.6	76.2	67.8	74.4
mIgG2a	-	70.2	71.4	75.0	71.4	74.4	66.6	73.8
mIgG2a	V1+C19	64.2	67.8	73.8	70.2	73.2	63.0	70.8

^1^ All antibodies listed in this table are homodimeric antibodies composed of a single HC and a single LC. They include the mFcγR binding-suppressing mutations shown in Figure 1 but do not contain the dKiHv14 mutations. When the FAST-Ig V1+C19 mutations are not present, it is denoted by “-”.

## Data Availability

Data are contained within the article.

## Supplementary Materials

The following supporting information can be downloaded at https://www.mdpi.com/article/10.3390/antib13010003/s1. Figure S1: Cation-exchange chromatography (CIEX) analysis for BsAbs with unmeasurable BsAb yields by mass spectrometry; Figure S2: Deconvoluted mass spectra of m2C4/mOKT3 with mIgG2a FAST-Ig V1+C19; Figure S3: Non-reducing SDS-PAGE analysis confirmed the effective formation of interchain disulfide bonds in BsAbs containing FAST-Ig V1+C19 mutations in both mIgG1 and mIgG2a formats; Figure S4: Size exclusion chromatography validates the high monomer ratio (>95%) of BsAbs with or without FAST-Ig V1+C19 mutations; Figure S5: SPR sensorgrams depicting the binding of anti-HER2 (H1L1H1L1) and anti-CD3 (H2L2H2L2) antibodies with or without FAST-Ig V1+C19 mutations to human HER2 and CD3, respectively. Table S1: The amino acid sequences of human CD3εδ used for the antigen preparation.
