# Supplementary material for: Rapid Generation of Murine Bispecific Antibodies Using FAST-IgTM for Preclinical Screening of HER2/CD3 T-Cell Engagers
Source: Antibodies (Basel). 2024 Jan 2;13(1):3. doi: 10.3390/antib13010003 (PMC10801562; doi:10.3390/antib13010003)
Supplement: Supplementary file 1 [file antibodies-13-00003-s001.zip › antibodies-2709504-supplementary.pdf]

Rapid Generation of Murine Bispecific Antibodies Using FAST-Ig™ for Preclinical Screening of HER2/CD3 T-Cell Engagers

Hikaru Koga *, Haruka Kuroi, Rena Hirano, Hiroyuki Hirayama, Yoshiaki Nabuchi and Taichi Kuramochi

Chugai Pharmaceutical Co., Ltd., Yokohama 244-8602, Japan; kuroi.haruka36@chugai-pharm.co.jp (H.K.); hirano.rena36@chugai-pharm.co.jp (R.H.); hirayama.hiroyuki37@chugai-pharm.co.jp (H.H.); nabuchiysa@chugai-pharm.co.jp (Y.N.); kuramochitic@chugai-pharm.co.jp (T.K.)

* Correspondence: koga.hikaru78@chugai-pharm.co.jp

Keywords: bispecific antibody; FAST-Ig; surrogate mouse bispecific antibody; orthogonal Fab; single-cell production

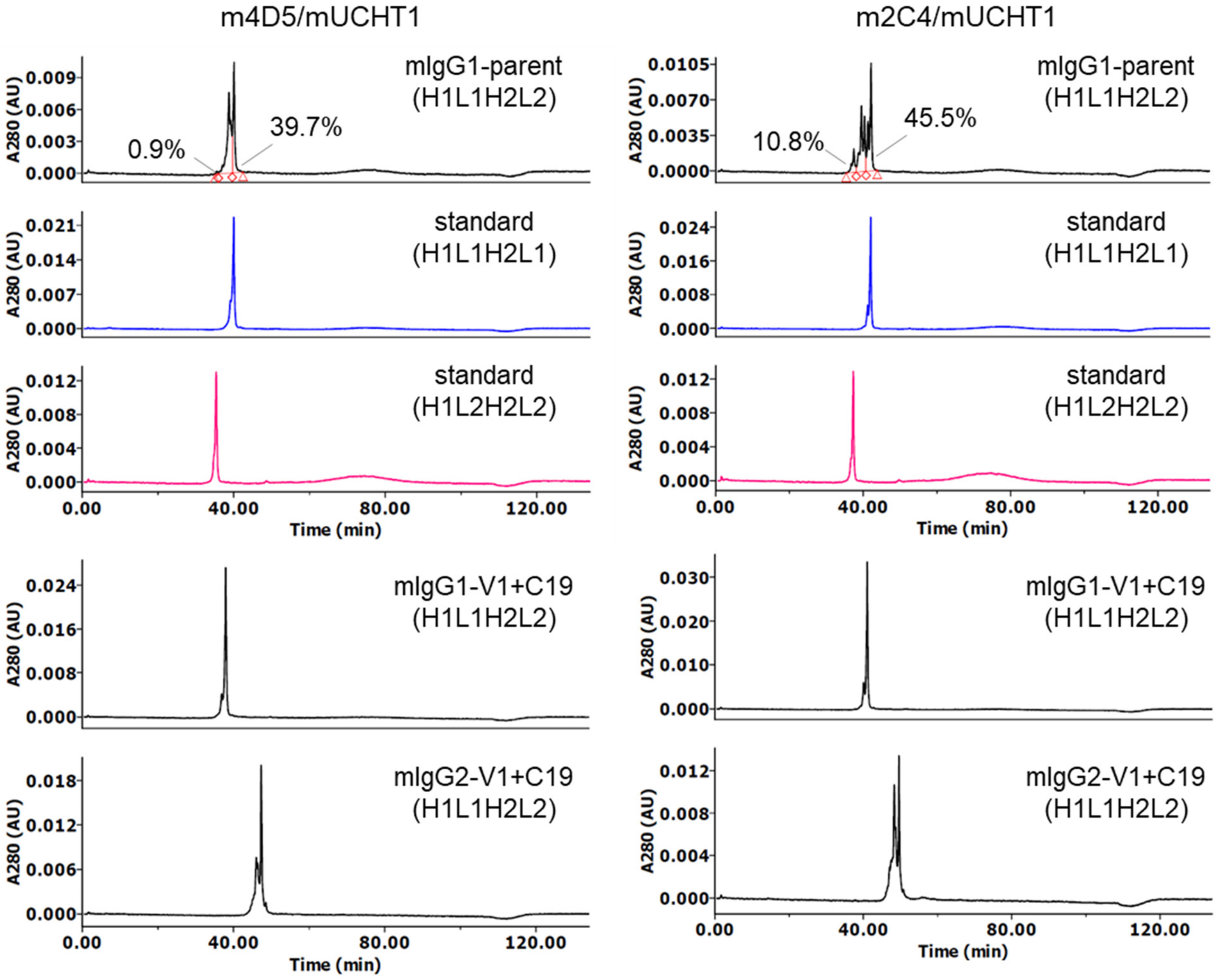

Figure S1. Cation-exchange (CIEX) chromatograms of BsAbs for which BsAb yields were not obtained by mass spectrometry. For the parent BsAbs, standard samples of H1L1H2L1 and H1L2H2L2 were prepared and analyzed to identify corresponding peaks. The area ratios of H1L1H2L1 and H1L2H2L2 for each sample are indicated in the chromatograms. The percentages of H1L1H2L2+H1L2H2L1 in the parent BsAbs of m4D5/mUCHT1 and m2C4/mUCHT1 were calculated to be 59.4% and 43.7%, respectively. In contrast, the introduction of FAST-Ig V1+C19 into the antibodies resulted in a lack of expression of H1L1H2L1 and H1L2H2L2 standard samples, implying a nearly 100% BsAb yield. Chromatograms of H1L1H2L2 only are shown for each sample. Sub-peaks in the vicinity of the main peak are considered to be charge variants of the main peak components.

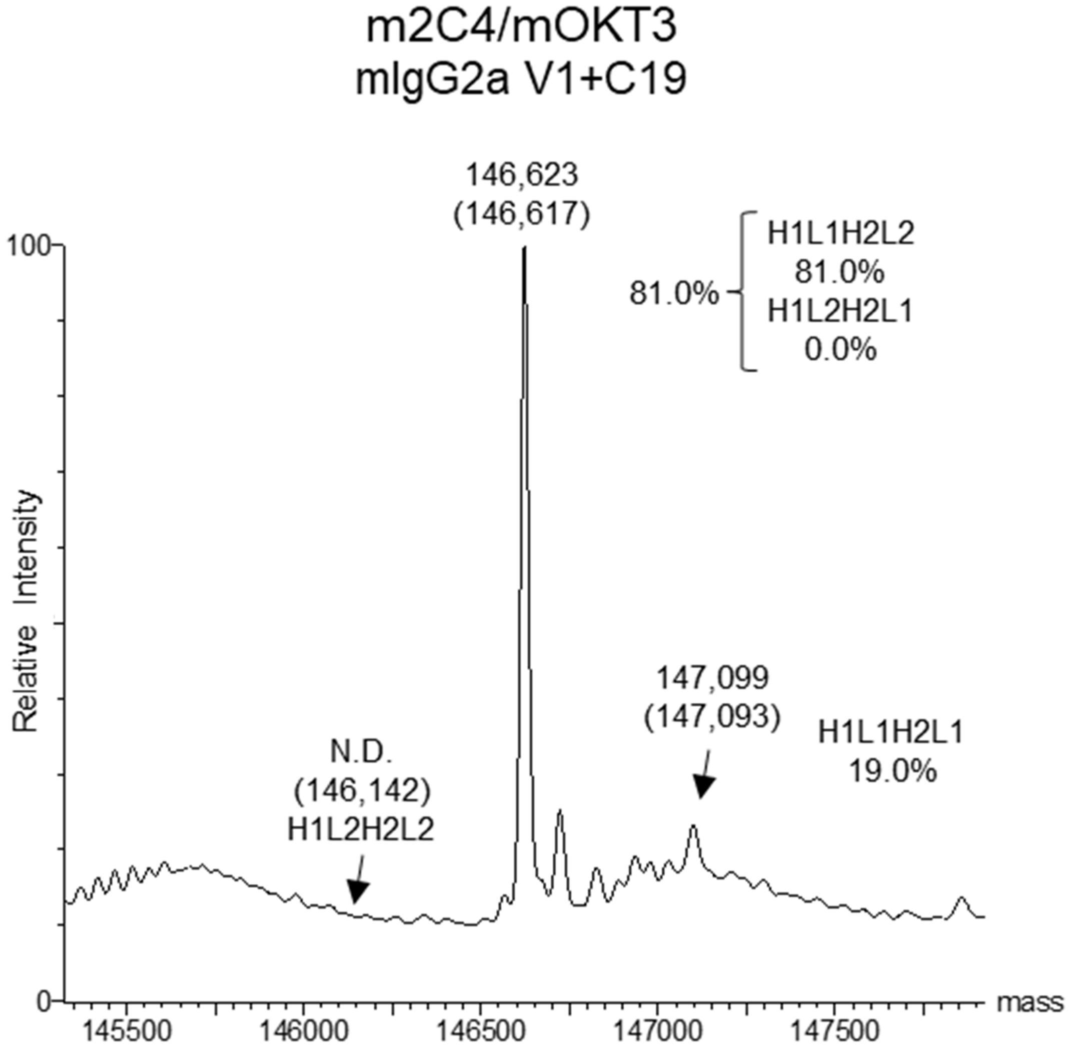

Figure S2. Deconvoluted mass spectrum of m2C4/mOKT3 with mIgG2a FAST-Ig V1+C19. The measured masses and theoretical masses (in parentheses) of each IgG species are labeled on each peak. The estimated percentages of the IgG species are also indicated. For this BsAb, due to the relatively high baseline noise, the percentage of mispair species (H1L1H2L1) might be estimated higher than the actual content.

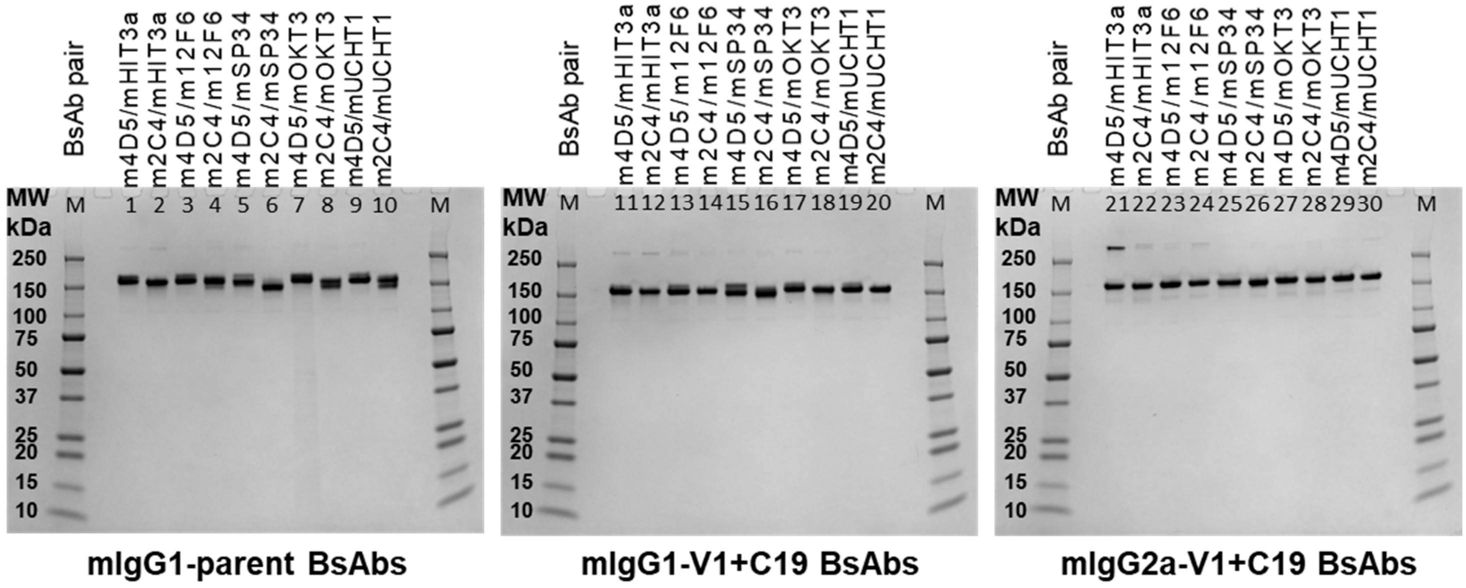

Figure S3. Non-reducing SDS-PAGE confirmed the formation of interchain disulfide bonds in BsAbs containing FAST-Ig V1+C19 mutations in both mIgG1 and mIgG2a formats.

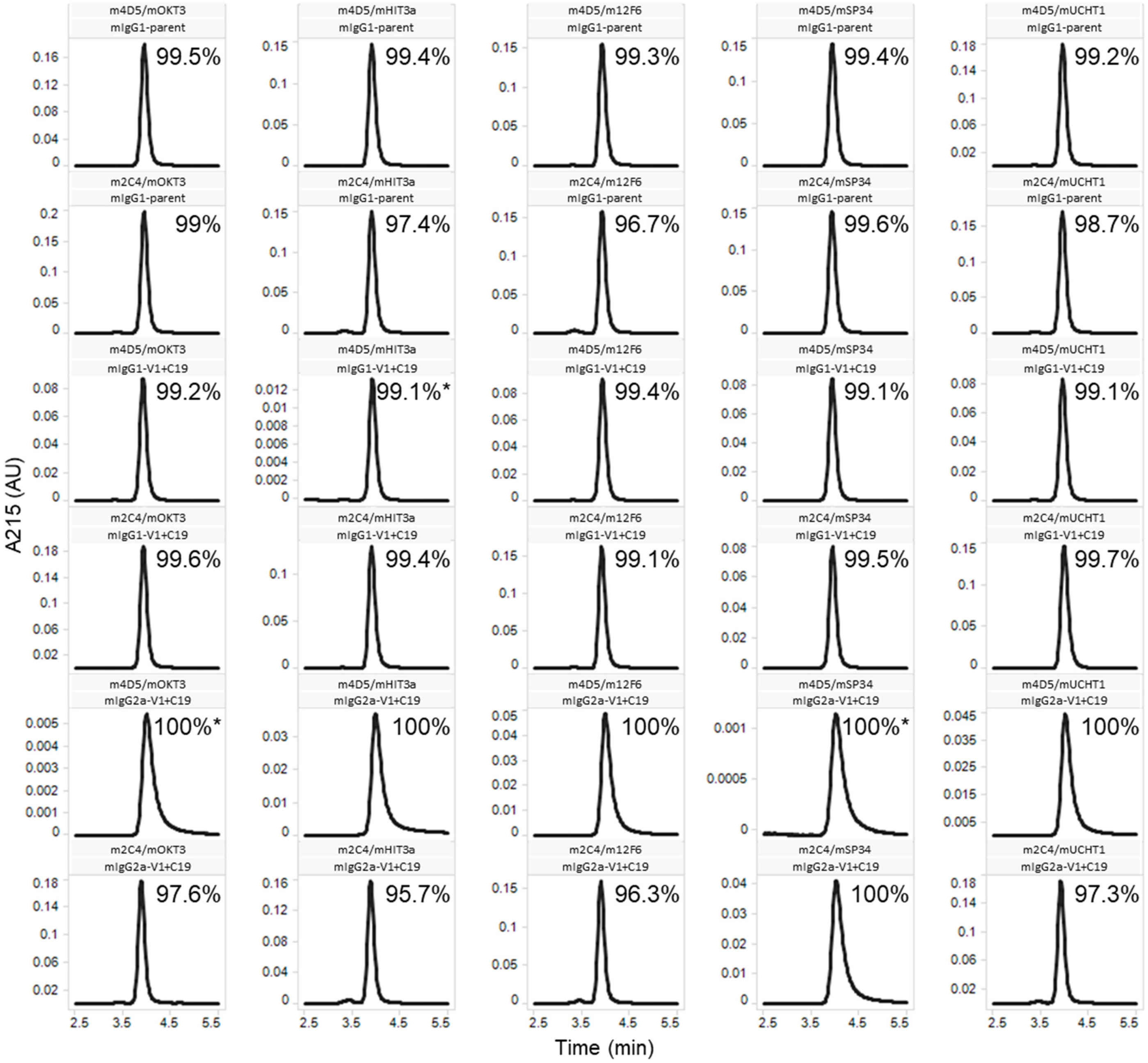

Figure S4. Size exclusion chromatography validates the high monomer ratio (>95%) of BsAbs with or without FAST-Ig V1+C19 mutations. Antibodies purified by protein A were analyzed. Ten μL of samples, diluted to either 0.05 or 0.1 mg/mL, were used for the analysis. The area percentage of the main peak in each chromatogram are shown in the chromatograms. Samples marked with an asterisk (*) had antibody concentrations lower than 0.05 mg/mL and were considered as roughly estimated values.

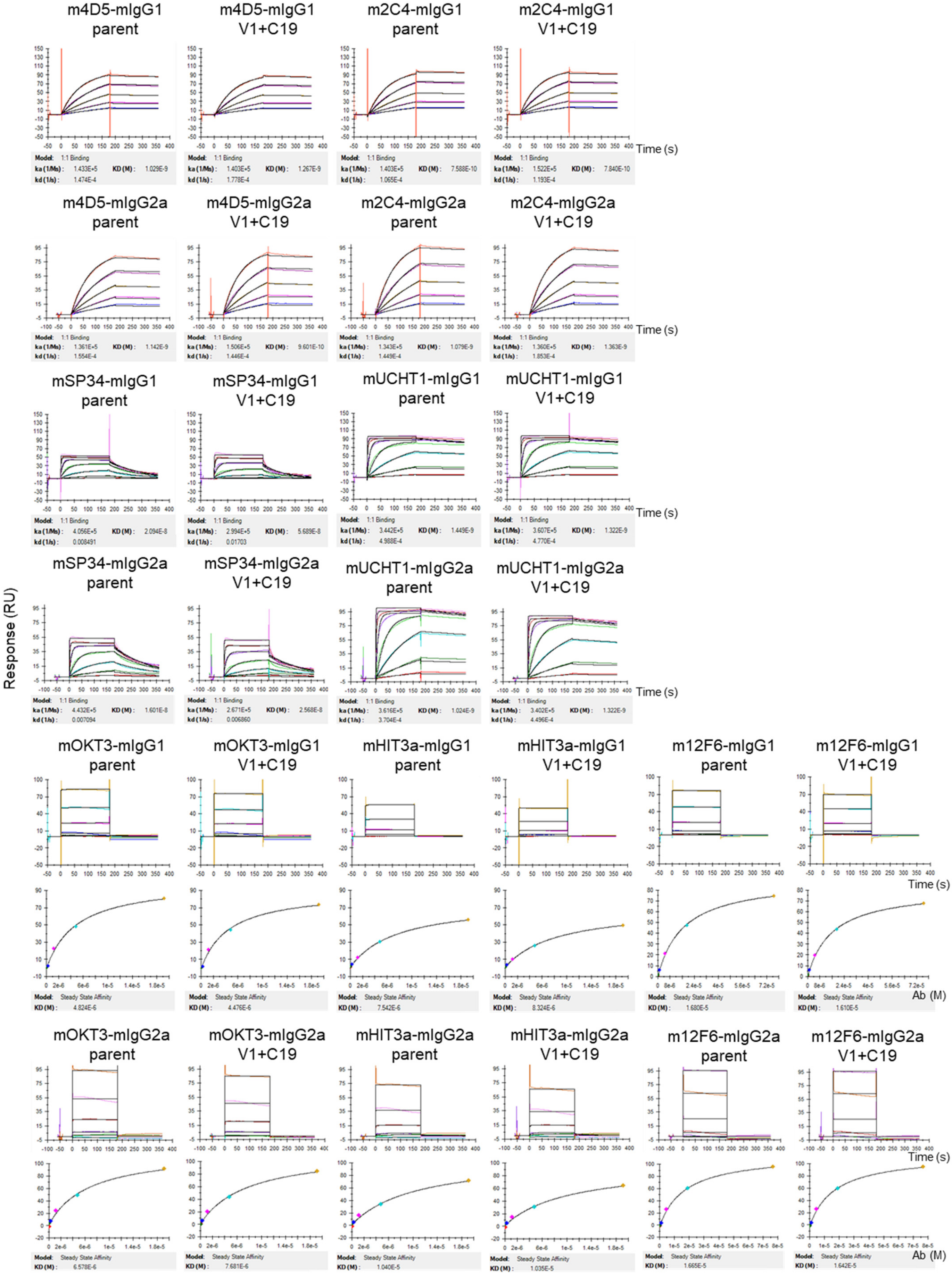

m4D5-mIgG1 parent
Model: 1:1 Binding
ka (1/Ms): 1.433E+5 KD (M): 1.029E-9
kd (1/s): 1.474E-4
m4D5-mIgG1 V1+C19
Model: 1:1 Binding
ka (1/Ms): 1.403E+5 KD (M): 1.267E-9
kd (1/s): 1.778E-4
m2C4-mIgG1 parent
Model: 1:1 Binding
ka (1/Ms): 1.403E+5 KD (M): 7.588E-10
kd (1/s): 1.065E-4
m2C4-mIgG1 V1+C19
Model: 1:1 Binding
ka (1/Ms): 1.522E+5 KD (M): 7.840E-10
kd (1/s): 1.193E-4
Time (s)
m4D5-mIgG2a parent
Model: 1:1 Binding
ka (1/Ms): 1.361E+5 KD (M): 1.142E-9
kd (1/s): 1.554E-4
m4D5-mIgG2a V1+C19
Model: 1:1 Binding
ka (1/Ms): 1.506E+5 KD (M): 9.601E-10
kd (1/s): 1.446E-4
m2C4-mIgG2a parent
Model: 1:1 Binding
ka (1/Ms): 1.343E+5 KD (M): 1.079E-9
kd (1/s): 1.449E-4
m2C4-mIgG2a V1+C19
Model: 1:1 Binding
ka (1/Ms): 1.360E+5 KD (M): 1.363E-9
kd (1/s): 1.853E-4
Time (s)
mSP34-mIgG1 parent
Model: 1:1 Binding
ka (1/Ms): 4.056E+5 KD (M): 2.094E-8
kd (1/s): 0.008491
mSP34-mIgG1 V1+C19
Model: 1:1 Binding
ka (1/Ms): 2.994E+5 KD (M): 5.689E-8
kd (1/s): 0.01703
mUCHT1-mIgG1 parent
Model: 1:1 Binding
ka (1/Ms): 3.442E+5 KD (M): 1.449E-9
kd (1/s): 4.988E-4
mUCHT1-mIgG1 V1+C19
Model: 1:1 Binding
ka (1/Ms): 3.607E+5 KD (M): 1.322E-9
kd (1/s): 4.770E-4
Time (s)
Response (RU)
mSP34-mIgG2a parent
Model: 1:1 Binding
ka (1/Ms): 4.432E+5 KD (M): 1.601E-8
kd (1/s): 0.007094
mSP34-mIgG2a V1+C19
Model: 1:1 Binding
ka (1/Ms): 2.671E+5 KD (M): 2.568E-8
kd (1/s): 0.006860
mUCHT1-mIgG2a parent
Model: 1:1 Binding
ka (1/Ms): 3.616E+5 KD (M): 1.024E-9
kd (1/s): 3.704E-4
mUCHT1-mIgG2a V1+C19
Model: 1:1 Binding
ka (1/Ms): 3.402E+5 KD (M): 1.322E-9
kd (1/s): 4.496E-4
Time (s)
mOKT3-mIgG1 parent
Model: Steady State Affinity
KD (M): 4.824E-6
mOKT3-mIgG1 V1+C19
Model: Steady State Affinity
KD (M): 4.476E-6
mHIT3a-mIgG1 parent
Model: Steady State Affinity
KD (M): 7.542E-6
mHIT3a-mIgG1 V1+C19
Model: Steady State Affinity
KD (M): 8.324E-6
m12F6-mIgG1 parent
Model: Steady State Affinity
KD (M): 1.680E-5
m12F6-mIgG1 V1+C19
Model: Steady State Affinity
KD (M): 1.610E-5
Time (s)
Ab (M)
mOKT3-mIgG2a parent
Model: Steady State Affinity
KD (M): 6.578E-6
mOKT3-mIgG2a V1+C19
Model: Steady State Affinity
KD (M): 7.681E-6
mHIT3a-mIgG2a parent
Model: Steady State Affinity
KD (M): 1.040E-5
mHIT3a-mIgG2a V1+C19
Model: Steady State Affinity
KD (M): 1.035E-5
m12F6-mIgG2a parent
Model: Steady State Affinity
KD (M): 1.665E-5
m12F6-mIgG2a V1+C19
Model: Steady State Affinity
KD (M): 1.642E-5
Time (s)
Ab (M)

Figure S5. SPR sensorgrams depicting the binding of anti-HER2 (H1L1H1L1) and anti-CD3 (H2L2H2L2) antibodies with or without FAST-Ig V1+C19 mutations to human HER2 and CD3, respectively. The antibody name and FAST-Ig mutations (or parent) are indicated above the sensorgrams. Colored lines represent binding response signals in a series of increasing antigen concentrations (as detailed in Materials and Methods). 1:1 kinetic model fits are overlaid in black. For mHIT3a, mOKT3, and m12F6, K_D (M) was calculated from the steady-state model due to very fast ka (1/Ms) and kd (1/s) rates.

Table S1. The amino acid sequences of human CD3εδ used for the antigen preparation.

Antigen	CD3ε-Flag	CD3δ-His
human CD3εδ	MQSGTHWRVLGLCLLSVGVWGQDGNEEM GGITQTPYKVSISGTTVILTCPQYPGSEILWQ HNDKNIGGDEDDKNIGSDEDHLSLKEFSELE QSGYYVCYPRGSKPEDANFYLYLRARVCEN CMEMDVMSDYKDDDDK	MEHSTFLSGLVLATLLSQVSPFKIPIEELE DRVFVNCNTSITWVEGTVGTLLSDITRLDL GKRILDPRGIYRCNGTDIYKDKESTVQVH YRMCQSCVELDPATVAGHHHHHHHH

The accession numbers of CD3ε and CD3δ are P07766 and P04234, respectively. Each extracellular domain with affinity tag sequence (Flag or His) was used.
